# Nuclear envelope impairment is facilitated by the herpes simplex virus 1 Us3 kinase

**DOI:** 10.12688/f1000research.17802.1

**Published:** 2019-02-18

**Authors:** Peter Wild, Sabine Leisinger, Anna Paula de Oliveira, Jana Doehner, Elisabeth M. Schraner, Cornel Fraevel, Mathias Ackermann, Andres Kaech

**Affiliations:** 1 Department of Veterinary Anatomy, University of Zuerich, Zürich, CH-8057, Switzerland; 2 Instute of Virology, University of Zürich, Zürich, ZH-8057, Switzerland; 3 Center for Microcopy and Image Analysis, Universit of Zürich, Zürich, CH-8057, Switzerland

**Keywords:** HSV-1 egress, nuclear pores, nuclear envelope breakdown, intraluminal transport, budding, fusion

## Abstract

**Background**: Capsids of herpes simplex virus 1 (HSV-1) are assembled in the nucleus, translocated either to the perinuclear space by budding at the inner nuclear membrane acquiring tegument and envelope, or released to the cytosol in a “naked” state via impaired nuclear pores that finally results in impairment of the nuclear envelope. The Us3 gene encodes a protein acting as a kinase, which is responsible for phosphorylation of numerous viral and cellular substrates. The Us3 kinase plays a crucial role in nucleus to cytoplasm capsid translocation. We thus investigate the nuclear surface in order to evaluate the significance of Us3 in maintenance of the nuclear envelope during HSV-1 infection.

**Methods**: To address alterations of the nuclear envelope and capsid nucleus to cytoplasm translocation related to the function of the Us3 kinase we investigated cells infected with wild type HSV-1 or the Us3 deletion mutant R7041(∆Us3) by transmission electron microscopy, focused ion-beam electron scanning microscopy, cryo-field emission scanning electron microscopy, confocal super resolution light microscopy, and polyacrylamide gel electrophoresis.

**Results**: Confocal super resolution microscopy and cryo-field emission scanning electron microscopy revealed decrement in pore numbers in infected cells. Number and degree of pore impairment was significantly reduced after infection with R7041(∆Us3) compared to infection with wild type HSV-1. The nuclear surface was significantly enlarged in cells infected with any of the viruses. Morphometric analysis revealed that additional nuclear membranes were produced forming multiple folds and caveolae, in which virions accumulated as documented by three-dimensional reconstruction after ion-beam scanning electron microscopy. Finally, significantly more R7041(∆Us3) capsids were retained in the nucleus than wild-type capsids whereas the number of R7041(∆Us3) capsids in the cytosol was significantly lower.

**Conclusions**: The data indicate that Us3 kinase is involved in facilitation of nuclear pore impairment and, concomitantly, in capsid release through impaired nuclear envelope.

## Introduction

Capsids of herpes simplex virus 1 (HSV-1) assemble in replication centers (RCs) in host cell nuclei ( Quinlan *et al.*, 1984). From there, they are transported to the nuclear periphery and are translocated to the cytoplasm via two diverse routes ( Roizman *et al.*, 2014). In one route, the nucleocytoplasmic barrier is overcome by budding of capsids at the inner nuclear membrane (INM). During budding, tegument and viral envelope are acquired ( Granzow *et al.*, 2001; Leuzinger *et al.*, 2005). The result is a fully enveloped virion located in the perinuclear space (PNS) delineated by the INM and outer nuclear membrane (ONM) that are part of the endoplasmic reticulum (ER). Virions in the PNS have been proposed for 5 decades to de-envelope by fusion of the viral envelope with the ONM releasing capsid and tegument into the cytoplasmic matrix ( Skepper *et al.*, 2001; Stackpole, 1969) for secondary envelopment at the trans Golgi network (TGN). Envelopment at the INM, de-envelopment at the ONM and re-envelopment at the TGN have been proposed to be essential for production of infectious progeny virus e.g. ( Mettenleiter *et al.*, 2006). The cruxes of the de-envelopment theory are i) that the viral envelope of Us3 deletion mutants cannot fuse with the ONM ( Wisner *et al.*, 2009), and hence, their capsids cannot be released into the cytoplasmic matrix. Consequently, they cannot be re-enveloped. Instead, virions of Us3 deletion mutants accumulate in the PNS. Despite of the inability of de- and re-envelopment Us3 deletion mutants are fully infective ( Reynolds *et al.*, 2002; Wild *et al.*, 2015; Wisner *et al.*, 2009). ii) The process taking place at the ONM exhibits all characteristics of budding shown for the first time 50 years ago ( Darlington & Moss, 1968). The process at the ONM also takes place in the absence of the fusion glycoproteins gB and gH leading to accumulation of virions in the PNS-ER compartment ( Farnsworth *et al.*, 2007). Therefore, the virus-membrane interaction taking place at the ONM is budding, indeed, not fusion as discussed in detail ( Wild *et al.*, 2018). Virions are transported out of the PNS into adjacent ER cisternae ( Gilbert *et al.*, 1994; Granzow *et al.*, 1997; Maric *et al.*, 2011; Radsak *et al.*, 1996; Schwartz & Roizman, 1969; Stannard *et al.*, 1996; Sutter *et al.*, 2012; Whealy *et al.*, 1991; Wild *et al.*, 2002). ER membranes connect to Golgi membranes forming a PNS-ER-Golgi continuum that is considered very likely to function as a direct intraluminal transportation route for virions from the PNS into Golgi cisternae ( Wild *et al.*, 2018).

Therefore, the question remains how naked capsids gain access to the cytoplasmic matrix if the viral envelope does not fuse with the ONM, and, consequently, de-envelopment does not take place. In cells infected with the monkey herpes pathogen simian agent 8 ( Borchers & Ozel, 1993), capsids gained access to the cytoplasmic matrix via impaired nuclear envelope (NE). It was clearly shown that the ONM turned into the INM at the sites of NE breakdown indicating that the NE breakdown was rather a result of nuclear pore impairment than a rupture of nuclear membranes. In bovine herpes virus 1 (BoHV-1) infected MDBK cells ( Wild *et al.*, 2005) and in HSV-1 infected Vero cells ( Leuzinger *et al.*, 2005; Wild *et al.*, 2009), impaired nuclear pores measured from about 150 nm to 300 nm. Large areas of impaired nuclear surface measuring several micrometers clearly exhibited intact transformation of the INM into the ONM indicating that NE impairment started by nuclear pore impairment. Impaired NE was also shown in cells infected with pseudorabies virus (PrV) UL31 and UL34-null recombinants ( Grimm *et al.*, 2012; Klupp *et al.*, 2011; Schulz *et al.*, 2015) as well as after HSV-1 infection of embryonic mouse fibroblasts ( Maric *et al.*, 2014). Capsids of HSV-1 and BoHV-1 were present in the nuclear matrix, which protruded through impaired nuclear pores into the cytoplasmic matrix, indicating that capsids are released via impaired NE. Capsids were also shown – though unrecognized – in impaired nuclear pores in HSV-1 infected mouse fibroblasts ( Maric *et al.*, 2014).

Us3 is a multifunctional protein that plays various roles in the viral life cycle by phosphorylating more than 20 viral and cellular substrates ( Kato & Kawaguchi, 2018). Phosphorylation of gB by Us3 was reported to be crucial for proper regulation of gB intracellular transport and in viral replication ( Imai *et al.*, 2011; Imai *et al.*, 2010). Us3 is involved in blocking apoptosis induced by HSV-1 ( Benetti *et al.*, 2003; Deruelle *et al.*, 2010; Jerome *et al.*, 1999; Leopardi *et al.*, 1997; Munger & Roizman, 2001; Ogg *et al.*, 2004), bovine herpes virus 1 ( Brzozowska *et al.*, 2018) and PrV ( Deruelle *et al.*, 2010). Us3 kinase is supposed to play a crucial role in capsid nucleus to cytoplasm translocation in association with phosphorylation of viral proteins including glycoprotein B ( Kato *et al.*, 2009; Wisner *et al.*, 2009), UL31 ( Mou *et al.*, 2009) and UL34 ( Ryckman & Roller, 2004). The 3 proteins facilitate translocation of virions out of the PNS ( Poon *et al.*, 2006; Reynolds *et al.*, 2004; Reynolds *et al.*, 2001; Reynolds *et al.*, 2002; Wisner *et al.*, 2009). In contrast, inhibited nucleus to cytoplasm translocation was suggested to be independent of phosphorylation of UL34 by Us3 in PrV infected cells ( Klupp *et al.*, 2001). UL31 and UL34 also promote the late maturation of viral replication compartments at the periphery ( Simpson-Holley *et al.*, 2004), and are involved in nuclear expansion during HSV-1 infection ( Simpson-Holley *et al.*, 2005). Us3 kinase also phosphorylates the nuclear lamin A/C ( Mou *et al.*, 2007) and is involved in disrupting the nuclear lamina together with UL34 ( Bjerke & Roller, 2006) possibly in association with phosphorylation of emerin ( Leach *et al.*, 2007). Recently, it was shown that UL31 and UL34 are responsible for budding of capsids at the INM ( Bigalke & Heldwein, 2015; Bigalke & Heldwein, 2016; Hagen *et al.*, 2015) and that the endosomal sorting complex required for transport-III (ESCRT III) is responsible for scission of the viral envelope from the INM ( Arii *et al.*, 2018). Us3 kinase down-regulates phospholipid biosynthesis ( Wild *et al.*, 2012a) induced by HSV-1 ( Sutter *et al.*, 2012) to maintain nuclear membrane integrity upon nuclear expansion and budding of capsids. Us3 kinase was suggested to inhibit breakdown of the NE ( Maric *et al.*, 2014).

Based on the proposed effects of Us3 kinase on the NE and nucleus to cytoplasm translocation we investigated the nucleus and the nuclear periphery in Vero cells infected with wild type (wt) HSV-1, the Us3 deletion mutant R7041(∆Us3) ( Purves *et al.*, 1987; Purves *et al.*, 1991) and its repair mutant R2641 by cryo-field emission scanning electron microscopy (cryo-FESEM) of cells after freezing and freeze-fracturing, by transmission electron microscopy (TEM) prepared by high pressure-freezing followed by freeze-substitution, and by super resolution light microscopy using the **sti**mulated **e**mission **d**epletion (STED) principle. The cryo-techniques enable visualization of structures in great detail, and, even more important, in a state that is closest to the situation in living cells ( Harreveld & Fifkova, 1975). The data suggest that Us3 kinase is involved in facilitation of nuclear pore impairment as well as in intranuclear capsid transportation and capsid release via impaired nuclear pores.

## Methods

### Cells and viruses

Vero cells (European Collection of Cell Cultures, ECACC, 84113001) were grown in Dulbecco’s modified minimal essential medium (DMEM, 31885-023; Gibco, Bethesda, MD, USA) supplemented with penicillin (100 U/ml), streptomycin (100 μg/ml) (Anti-Anti, 15240-062, Gibco) and 10% fetal bovine serum (FBS; 2-01F10-I, Bio Concept, Allschwil, Switzerland). Wild-type (wt) strain F ( Ejercito *et al.*, 1968), the Us3 deletion mutant R7041(∆Us3) and the repair mutant R2641 (kindly provided by B. Roizman, The Marjorie B. Kovler Viral Oncology Laboratories, University of Chicago, Illinois, USA). Wt HSV-1 were propagated in Vero cells. Virus yields were determined by plaque titration. For infection, cells were washed with DMEM without FBS, inoculated with virus diluted in DMEM without FBS, and kept for 1 h at 37°C. Then, cells were quickly washed with PBS, and incubated at 37°C in the presence of DMEM supplemented with 2%FBS. For controls, cells were mock infected by the same procedure replacing virus suspension with DMEM without FBS.

### Cryo-fixation for transmission electron microscopy and focused ion beam scanning electron microscopy (FIB-SEM)

50 mm thick sapphire disks (100.00174, Bruegger, Minusio, Switzerland) measuring 3 mm in diameter were coated with 8–10 nm carbon obtained by evaporation under high vacuum conditions to enhance cell growth. Vero cells were grown for 2 days on sapphire disks placed in 6 well plates. Cells were inoculated with R7041(∆Us3), the repair mutant R2641 or wt HSV-1 at a multiplicity of infection (MOI) of 5, incubated at 37°C, and fixed at 9, 12, 16, 20 and 24 hours post infection (hpi) by adding 0.25% glutaraldehyde to the medium prior to freezing in a high-pressure freezing unit (HPM010; BAL-TEC, Balzers, Lichtenstein) and processed as described in detail ( Wild, 2008). In brief, the frozen water was substituted with acetone in a freeze-substitution unit (FS 7500; Boeckeler Instruments, Tucson, AZ, USA) at -88°C, and subsequently fixed with 0.25% glutaraldehyde and 0.5% osmium tetroxide (in water) raising the temperature gradually to +2°C to achieve good contrast of membranes ( Wild *et al.*, 2001), and embedded in epon prepared by mixing 61g Epon 812 (45345, Merck, Darmstadt, Germany), 40g Dodecenylsuccinic anhydride (DDSA, 45346, Merck), 27g methyl nadic anhydride (MNA, 45347, Merck) and 1.92 ml 2,4,6-Tris(dimethylaminomethyl)phenol (DMP30, 45348, Merck) at 4°C followed by polymerization at 60°C for 2.5 days. Serial sections of 60 to 90 nm thickness were analyzed in a transmission electron microscope (CM12; FEI, Eindhoven, The Netherlands) equipped with a CCD camera (Ultrascan 1000; Gatan, Pleasanton, CA, USA) at an acceleration voltage of 100 kV.

For 3D reconstruction, a trimmed epon block was mounted on a regular SEM stub using conductive carbon and coated with 10 nm of carbon by electron beam evaporation to render the sample conductive. Ion milling and image acquisition was performed simultaneously in an Auriga 40 Crossbeam system (Zeiss, Oberkochen, Germany) using the FIBICS Nanopatterning engine (NPVE v4.6, Fibics Inc., Ottawa, Canada). A large trench was milled at a current of 16 nA and 30 kV, followed by fine milling at 240 pA and 30 kV during image acquisition with an advance of 5 nm per image. Prior to starting the fine milling and imaging, a protective Platinum layer of approximately 300 nm was applied on top of the surface of the area of interest using the single gas injection system at the FIB-SEM. Images were acquired at 1.9 kV (30 µm aperture) using an in-lens energy selective backscattered electron detector (ESB) with a grid voltage of 500 V, and a dwell time of 1 μs and a line averaging of 50 lines. The pixel size was set to 5 nm and tilt-corrected to obtain isotropic voxels. The final image stack was registered and cropped to the area of interest for segmentation using the TrakEM2 plug-in (version 1.0i) for Fiji image-processing package v1.51f.

### Cryo-field emission scanning electron microscopy (Cryo-FESEM)

Vero cells were grown in 25 cm ^2^ cell culture flasks for 2 days prior to inoculation with R7041(∆Us3), wt HSV-1 or R2641 at MOI of 5. Cells were harvested at 16 hpi by trypsinization followed by centrifugation at 150 × g for 8 min. The pellet was resuspended in 1 ml fresh medium, collected in Eppendorf tubes and fixed by adding 0.25% glutaraldehyde to the medium. The suspension was kept in the tubes at 4°C until cells were sedimented. After removal of the supernatant cells were frozen in a high-pressure freezing machine EM HPM100 (Leica Microsystems, Vienna, Austria) as described in detail previously ( Wild *et al.*, 2012b; Wild *et al.*, 2009). Cells were fractured at -120°C in a freeze-fracturing device BAF 060 (Leica Microsystems) in a vacuum of 10 ^-7^ mbar. The fractured surfaces were partially freeze-dried (“etched”) at -105°C for 2 min, and coated with 2.5 nm platinum/carbon by electron beam evaporation at an angle of 45°. Some specimens were coated additionally with 4 nm of carbon to reduce electron beam damage during imaging at high magnifications. Specimens were imaged in an Auriga 40 Cross Beam system equipped with a cryo-stage (Zeiss, Oberkochen, Germany) at -115°C and an acceleration voltage of 5 kV using the inlens secondary electron detector.

### Confocal microscopy

Cells were grown for 2 days on 0.17 mm thick cover slips measuring 12 mm in diameter (Hecht-Assistent, Sondheim, Germany) and inoculated with R7041(∆Us3), wt HSV-1 or R2641 at a MOI of 5 and incubated at 37°C. After fixation with 2% formaldehyde for 25 min at room temperature, cells were permeabilized with 0.1% Triton-X-100 at room temperature for 7 min and blocked with 3% bovine serum albumin in phosphate-buffered saline containing 0.05% Tween 20 (PBST20). To identify nuclear pore complexes, cells incubated for 16 h were processed as described ( Wild *et al.*, 2009) using mouse monoclonal antibodies Mab414 (MMS-120P, Covance, Princeton, NJ, USA), and Alexa 488-conjugated secondary antibodies (goat anti-mouse, A32723, Thermo Fisher, Rockford, IL, USA). To identify infectivity, cells were labeled with polyclonal antibodies (1:1000) raised in rabbits against the tegument protein VP16 (gift from B. Roizman), and with Alexa 594-conjugated secondary antibodies, diluted 1:500, (goat anti-rabbit, A11037, Thermo Fisher Scientific). For measuring nuclear diameters, nuclei were stained with 4’,6’-diamidino-2-phenylindole (DAPI). Cells were embedded in glycergel mounting media (C0563, Dako North America, Carpinteria, CA, USA) and 25 mg/ml DABCO (1,4-diazabicyclo [2.2.2] octane; 33480, Fluka, Buchs, Switzerland). Specimens were analyzed using a confocal laser scanning microscope (SP2, Leica, Microsystems, Wetzlar, Germany).

 For super-resolution imaging, DAPI staining was avoided, Alexa 532-conjugated antibodies (goat anti mouse, 1:500) were used as secondary antibodies for Mab414, and Alexa 488 as secondary antibodies for VP16. Cells were mounted with ProLong Gold Antifade Reagent (P36930 Thermo Fisher). Images were acquired with a TCS SP8 gSTED 3X microscope (Leica Microsystems, Wetzlar, Germany), which allows, in addition to standard confocal microscopy, the use of the gated STED (gSTED) principle to perform imaging beyond the diffraction limit. An HC PL Apo STED White 100x/1.4NA oil objective was used to obtain super resolved images with a final pixel size of 20 nm. The nuclear pores were excited using a super continuum white light laser (WLL) at a wavelength of 532 nm, depleted with a STED laser beam at 660 nm and detected with hybrid detectors adapted for time gated imaging (applied time gate: 1.5 – 7 ns). For analysis, the images were deconvolved employing the deconvolution algorithm of the program suite Huygens Professional version 18.04 (SVI, Hilversum, The Netherlands).

### Morphometric analysis

Nuclei of Vero cells are triaxial ellipsoids. Therefore, the mean nuclear volume (V _n_) and mean nuclear surface area (S _n_) were calculated on the basis of the half axes (a, b, c) measured on 25 deconvolved confocal images of DAPI stained nuclei as described in detail recently ( Sutter *et al.*, 2012). Capsids within nuclei were counted on TEM images selected at random at 16 hpi). Then the nuclear area was estimated by point counting applying a multipurpose test system ( Weibel, 1979). The mean nuclear area (A _n_) was calculated using the equation A _n_ = P _n_·d ^2^, whereby P _n_ are points hitting the nuclei and d the test line length. Capsids were counted on nuclear profiles. From the number of capsids (c) and the nuclear area, the numerical density N _Vc_ = c/(A _n_)/D can be calculated, whereby D is the mean particle diameter: D =125 nm for capsids ( Zhou *et al.*, 1998). Then, the total number of capsids (N _c_) per mean nuclear volume can be calculated: N _c_= N _Vc_·V _n_. The mean number of RCs was expressed per nuclear profile because the true size of RCs cannot be measured accurately. Diameters of nuclear pores visualized by cryo-FESEM imaging were measured using the AnalySIS (version 5) Five software (Olympus, Hamburg, Germany). The number of nuclear pores were counted and expressed per 1 µm ^2^ nuclear area and calculated per the mean nuclear surface obtained from confocal images. The number of nuclear pore complexes (NPC) was determined on Mab414 stained nuclei using AnalySIS Five (Olympus).

To determine changes in nuclear membranes arising during R7041(∆Us3) infection, and the amount of membranes used for envelopment during budding, images were collected at a final magnification of 87500x. On these images the surface density of membrane folds (Sv _f_) and of the viral envelope in the PNS (Sv _e_) were estimated using the equations Sv _f,,e_ = 4I _f,e_/d·P _n_, whereby I _f,e_ are the number of intersections of the test lines d with membrane folds and viral envelope, respectively. From the surface density, the area of membrane folds and viral envelope were calculated per mean nuclear volume: S _f_= Sv _f_·V _n_ and S _e_= Sv _e_·V _n_. Mean and variance of nuclear pore diameters were compared by the Welch-Test, Mean and standard deviation of all data by a multiple t-test using GraphPad Prism version 8.

### Polyacrylamide gel electrophoresis and immunoblotting

Vero cells were grown in 25 cm ^2^ cell culture flasks. Cells were inoculated with R7041(∆Us3) or wt HSV-1 at a MOI of 5 and incubated at 37°C for 24 h. The protein extraction was accomplished as following. After washing with PBS protein lysis buffer (0.5 M Tris-HCl pH 6.8, 4.4% SDS, 1% β-mercaptoethanol, 20% glycerol, 1% bromphenol blue, H _2_O) was added, and the samples were boiled for 5 min. 10 µl protein of each sample were separated on 7% SDS-polyacrylamid gel. After electrophoresis at 100 V for 2 h, the proteins were blotted onto a nitrocellulose membrane (10600002, Amersham Biosciences Europe, Freiburg, Germany). Blots were blocked with 5% low-fat milk in PBST20 (50 mM sodium phosphate buffer containing 155 mM NaCl and 0.3% Tween 20) over night. Subsequently, blots were probed with monoclonal mouse antibodies against capsid protein ICP5 (ab6508, Abcam, Cambridge, UK), diluted 1:3200, and polyclonal antibodies against tegument proteins VP16 and VP22 raised in rabbits (gift from B. Roizman), diluted in PBST20 (1:3000 to 1:5000). After two washing steps with PBST20, blots were incubated with horse radish peroxidase-conjugated anti-mouse, diluted 1:10000, (AP124P, Sigma-Aldrich, Buchs, Switzerland) or anti-rabbit secondary antibodies, diluted 1:1000, (GERPN4301, Sigma-Aldrich). Protein bands were visualized on X-ray films using chemiluminescence. For loading control, antibodies were stripped out of the membranes with Restore Western blot stripping buffer (21059, Thermo Fisher Scientific) according to manufacturer instructions. Membranes were probed with monoclonal anti-beta actin antibodies produced in mouse, diluted 1:1000 (SAB1305567, Sigma-Aldrich).

## Results

### HSV-1 induced nuclear pore impairment is reduced in the absence of Us3

**Figure 1.**
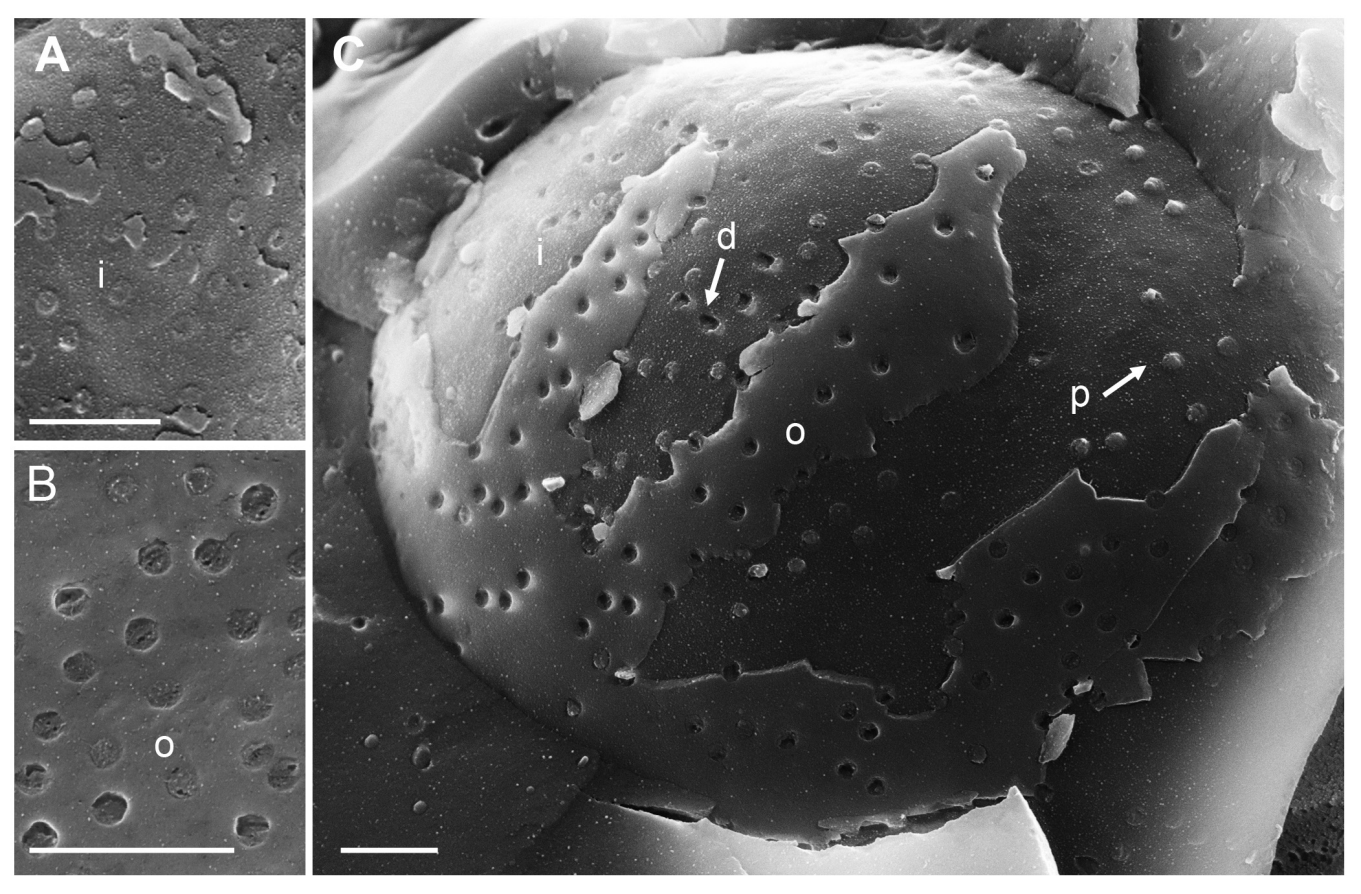
Cryo-field emission scanning electron microscopy (cryo-FESEM) of mock infected cells. ( **A**) Detail of inner nuclear membrane (INM) (i), ( **B**) detail of outer nuclear membrane (ONM) (o) showing nuclear pore complex (NPC) anchored within the nuclear pore. ( **C**) Overview showing in addition to nuclear pores with anchored NPC, pores of which the NPC has been removed (d) as well as pores with protruding NPC (p). Bars 500 nm.

**Figure 2.**
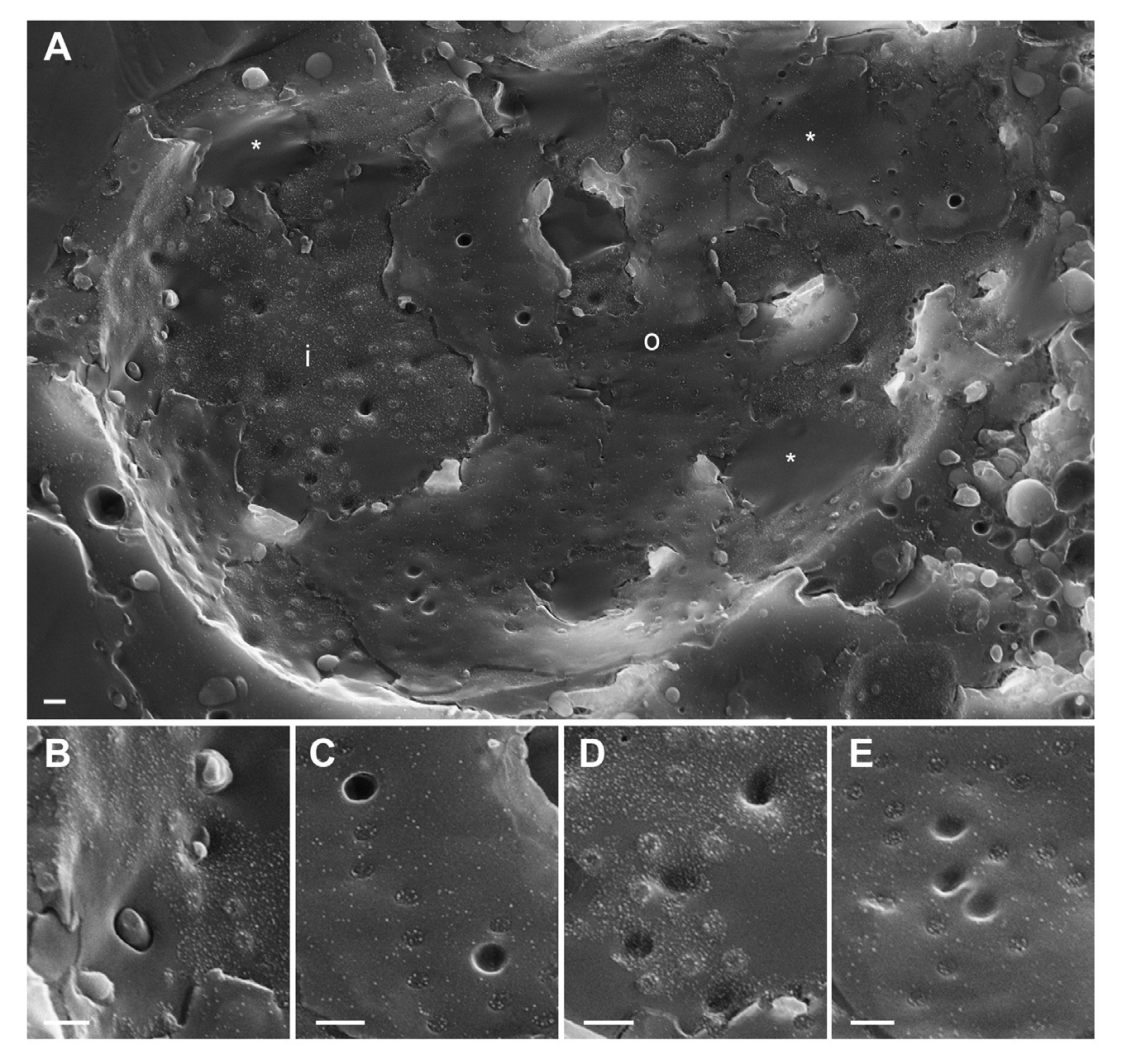
The nuclear surface of Vero cells imaged 14 hpi with wt HSV-1 by cryo-FESEM showing in panel ( **A**) large areas devoid of nuclear pores (asterisks) at the inner nuclear membrane (INM) (i) and outer nuclear membrane (ONM) (o), holes with ( **B**) or without ( **C**) protruding material, large depressions at the INM ( **D**) and at the ONM ( **E**). Bars 200 nm.

**Figure 3.**
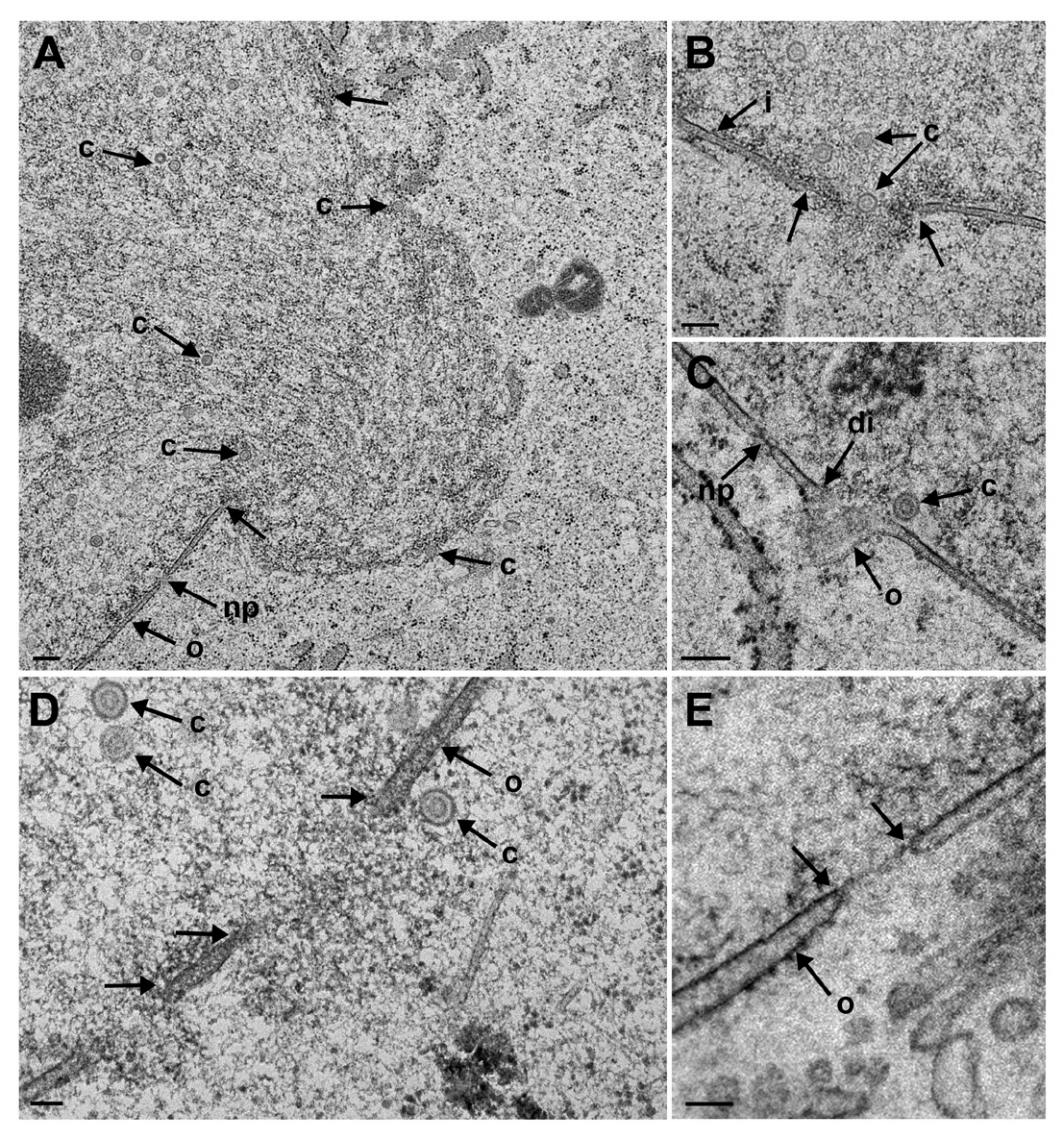
Transmission electron microscopy (TEM) images of dilated nuclear pores at 16 hpi ( **A** to **D**) and at 12 hpi ( **E**) with wt HSV-1. The outer nuclear membrane (ONM) (o) continues into the inner nuclear membrane (INM) (i) clearly visible (arrows) at least at one side ( **A**, **B** and **D**). The nuclear material protruding into the cytoplasm contains capsids (c) indicating that capsids are released into the cytoplasm. Possible breakdown of nuclear membrane ( **C**). The INM is disrupted (di), and the ONM runs towards the cytoplasm just beside an intact nuclear pore (np). Nuclear pore dilated to 170 nm distinctly showing the continuum (arrows) between ONM and INM ( **E**). Bars 200 nm ( **A**, **B**, **C**), 100 nm ( **D**, **E**).

To visualize the nuclear surface in the frozen hydrated state, frozen cells need to be fractured. Fracturing of frozen hydrated cells does not create completely arbitrary surfaces. Rather, fracture planes run preferentially along the hydrophobic center of the lipid bilayer of cell membranes, e.g. along the center of the INM or ONM ( Severs, 2007). In cryo-FESEM images, intact nuclear pores appear basically as flat button-like structures at the INM, and as small indentations at the ONM ( Figure 1) as described in detail ( Wild *et al.*, 2012b) and by many other authors from the early days of introducing the freeze-fracture technique, e.g. ( Haggis, 1989; Nicolini *et al.*, 1984; Teigler & Baerwald, 1972). Nuclear pore diameter measures 125 nm in negatively stained frog oocytes (Pante & Aebi, 1996). The diameter of nuclear pores in mock infected Vero cells imaged by cryo-FESEM varies due to changes taking place during preparation and imaging. The NPC can be removed together with the ONM during cryo-fracturing leading to small depressions at the INM. Alternatively, the NPC may slightly protrude into the cytoplasm ( Wild *et al.*, 2012b). The average diameter of these small protrusions was 120 nm. Distribution of nuclear pores was irregular ( Figure 1C). In wt HSV-1 infected cells, large areas of the nuclear surface were devoid of nuclear pores and of nuclear membrane proteins ( Orci & Perrelet, 1975). This was also shown in herpes virus infected BHK-21 cells employing the freeze-fracture technique ( Haines & Baerwald, 1976). Most of the nuclear pores appeared similar as in mock infected cells ( Figure 2). However, there were large clearly confined holes. Many of the holes contained material protruding into the cytoplasm. TEM analysis revealed that most of these holes were confined by an intact INM turning into the ONM ( Figure 3A and B), and that nuclear material containing capsids protruded through the holes into the cytoplasm. In a sole case, the nuclear membranes were obviously disrupted ( Figure 3C). We thus conclude that the clearly confined holes are dilated nuclear pores.

**Figure 4.**
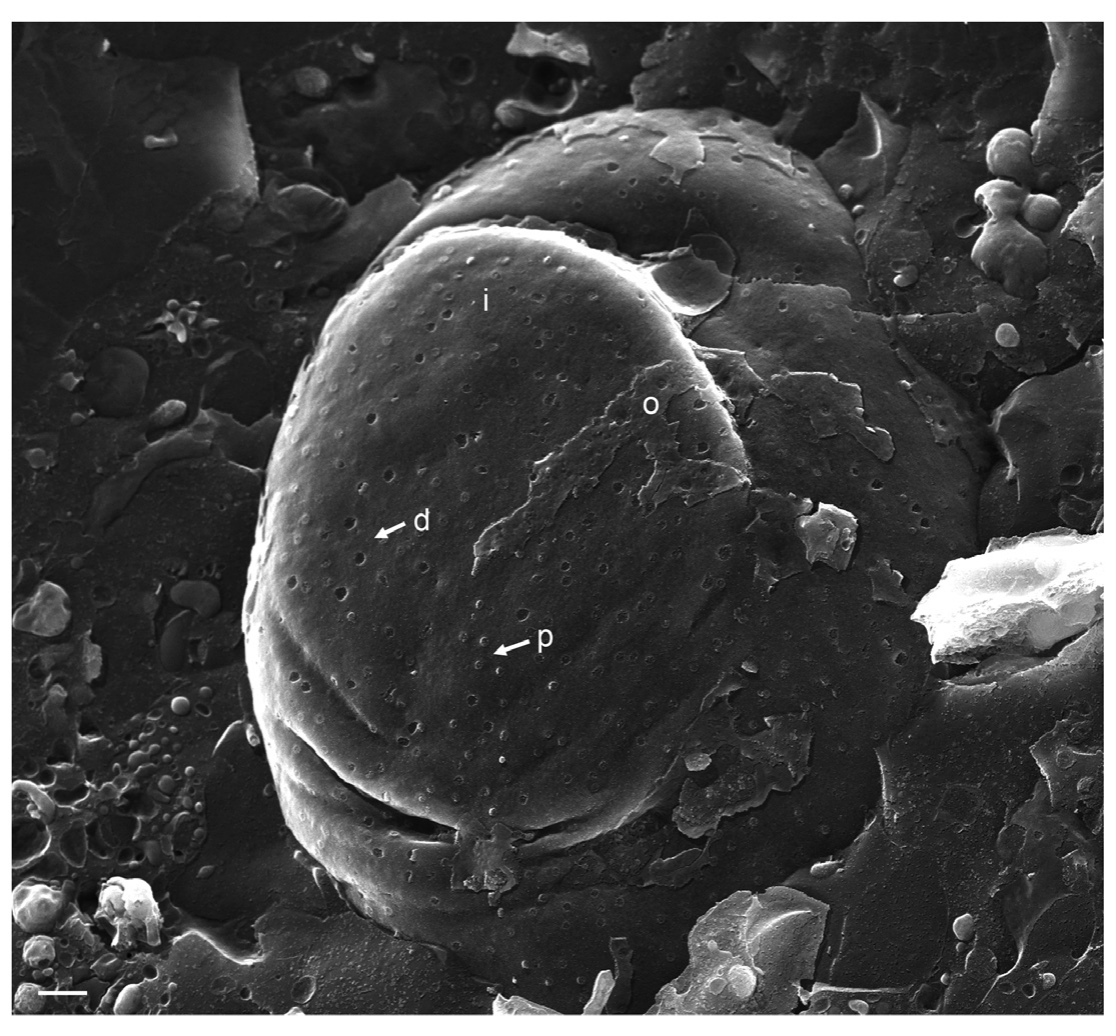
Cryo-field emission scanning electron microscopy (cryo-FESEM) of a Vero cell at 16 hpi with R7041(∆Us3). The nuclear surface is folded and invaginated. It contains several nuclear pores of which nuclear pore complex (NPC) has been removed during fracturing (d) or with slightly protruding NPC (p) at the inner nuclear membrane (INM) (i). The outer nuclear membrane (ONM) (o) has been largely removed. Bar 500 nm.

**Figure 5.**
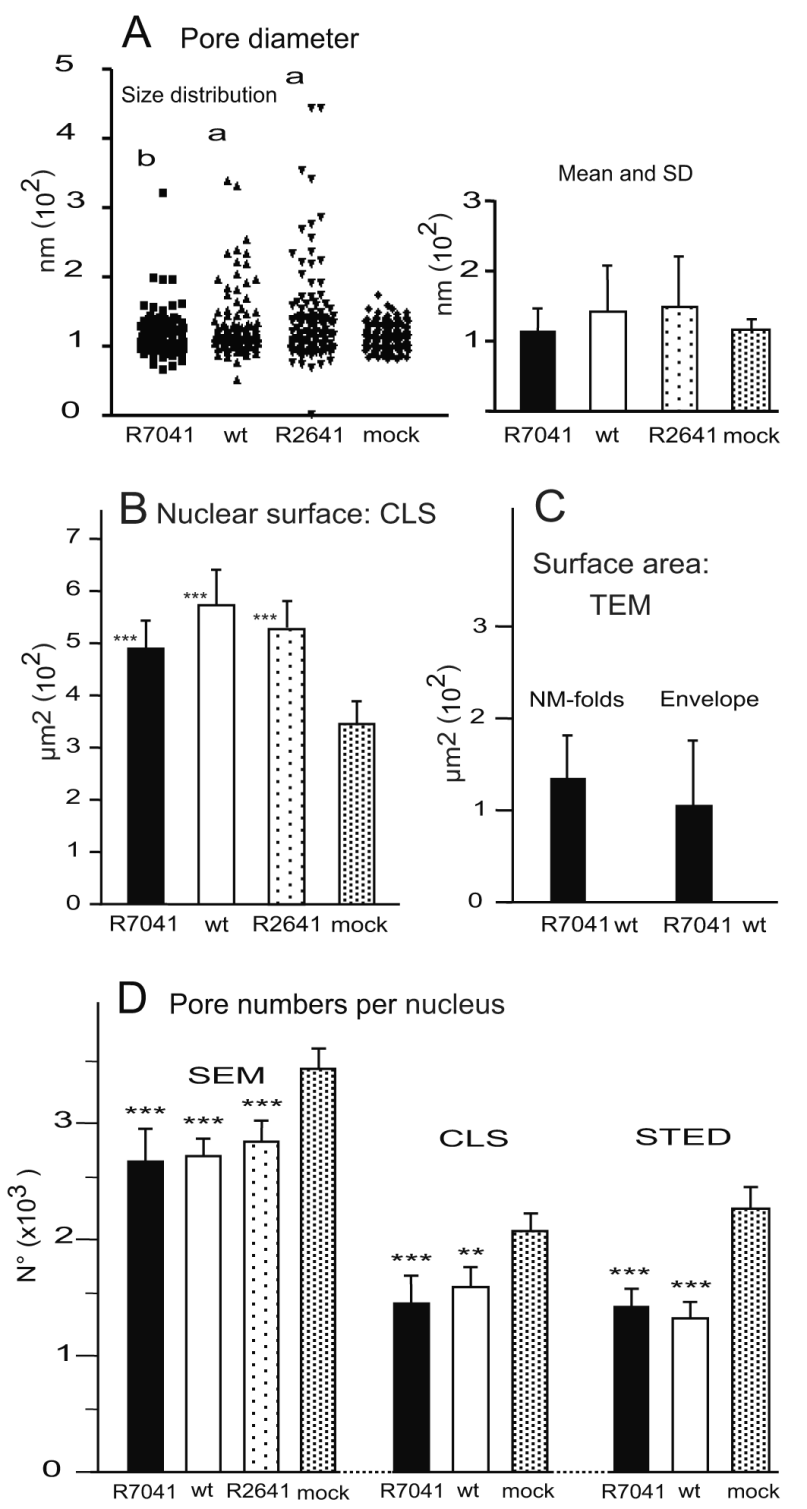
Number and size of nuclear pores determined on 10 nuclei at 16 hpi with R7041(∆Us3), the repair mutant R2641 or wt HSV-1, and of mock infected cells. ( **A**) Range of nuclear pore diameter, as well as mean and SD. Level of significance a) p<0.0001 for variance and mean diameter compared to R7041(∆Us3) or mock, b) p=0.89 for the mean diameter compared to mock, n=10. ( **B**) Mean nuclear surface area. ( **C**) Surface area of membrane folds and of the total number of virions present in the perinuclear space (PNS) at 16 hpi with R7041(∆Us3) and HSV-1 that was close to zero. (D) Number of nuclear pores counted on cryo-field emission scanning electron microscopy (cryo-FESEM) images and calculated per mean nuclear surface area or determined on confocal microscopic (CLS) or stimulated emission depletion (STED) images after labeling of pore complexes with Mab414. Level of significance **p<0.01, ***p<0.001 compared to mock; n=10 (STED: n=3).

In cells infected with the deletion mutant R7041(∆Us3), the most striking feature was the irregular nuclear surface showing folds and invaginations ( Figure 4). Nuclear pores appeared similar as in mock-infected cells. The number of dilated pores was low. To address frequency and size of pore dilation, we measured nuclear pores on 10 nuclei harvested at 16 h post inoculation (hpi). In mock infected cells, pore diameter ranged between 90 and 140 nm with a few exceptions ( Figure 5A). In cells infected with R7041(∆Us3), nuclear pores measured up to 180 nm, and in wt HSV-1 or the Us3 repair mutant R2641 up to 400 nm. The mean pore diameter was significantly larger (p<0.0001) in wt HSV-1 and R2641 infected cells compared to mock or R7041(∆Us3) infected cells whereas it did not differ significantly between R7041(∆Us3) and mock infection. The variance of pore diameter was significantly different (p<0.0001) between all groups except between wt HSV-1 and R2641 infected cells. NPCs disintegrate and, subsequently, nuclear pores dilate in the course of NE breakdown during mitosis ( Georgatos *et al.*, 1997; Terasaki *et al.*, 2001). HSV-1 arrests the cell cycle in the G1/S phase and S phase ( de Bruyn Kops & Knipe, 1988; Ehmann *et al.*, 2000). Mitotic activity almost completely declines by 6 hpi with wt HSV-1 at a MOI of 5 ( Sutter *et al.*, 2012). Therefore, pore dilation in HSV-1 infected cells is likely not related to mitosis. In measuring pore diameter, we only considered nuclei with clear indications of infection such as budding capsids. We thus conclude that i) nuclear pores dilate during HSV-1 infection, and ii) dilation of nuclear pores is facilitated by the Us3 kinase.

### Nuclear membranes expand during R7041(∆Us3) infection

**Figure 6.**
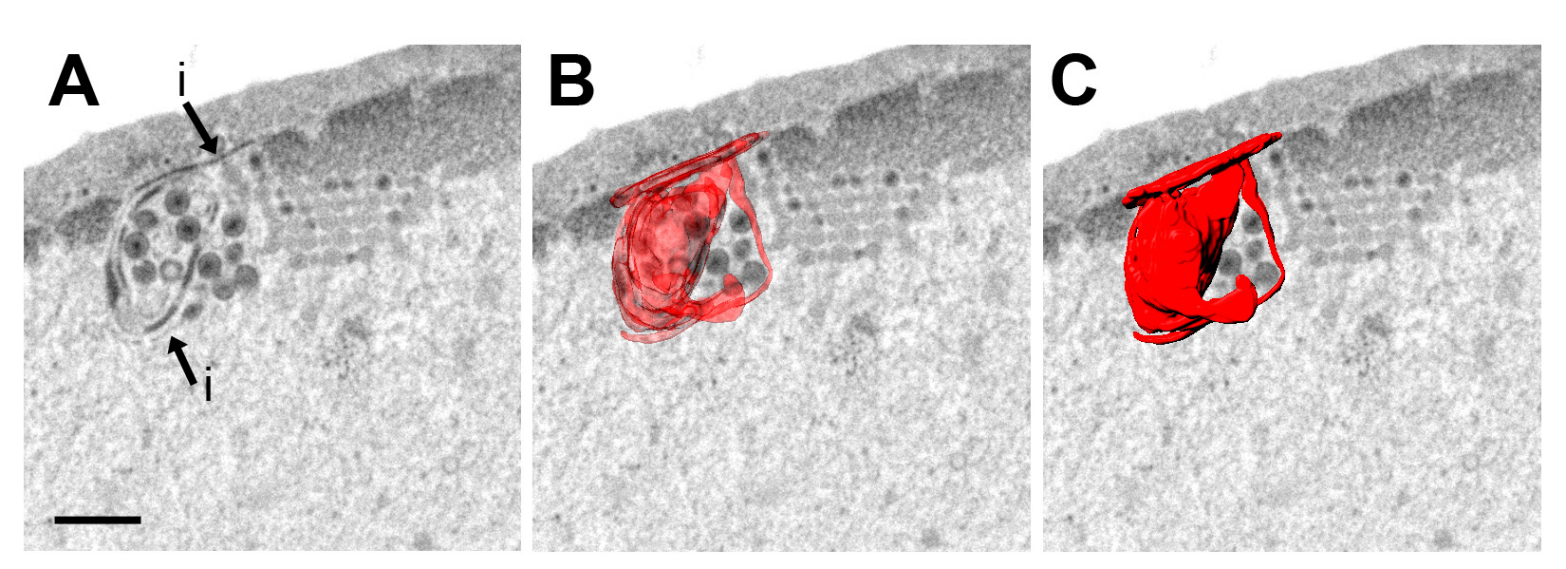
Focused ion beam scanning electron microscopy (FIB-SEM) image and 3D reconstruction of folds of the inner nuclear membrane (INM) (i) enclosing cavities in which virions accumulate. Bar 500 nm.

The nuclear membranes expand during infection with HSV-1. Expansion of nuclear membranes is out of control in cells infected with Us3 deletion mutants leading to formation of folds and invagination that contain virions ( Reynolds *et al.*, 2002; Wild *et al.*, 2012a; Wild *et al.*, 2015; Wisner *et al.*, 2009). We calculated the excessively produced membranes by morphometric analysis. The membrane folds cover an area equaling about 30% of the total nuclear surface area ( Figure 5C) at16 hpi with R7041(∆Us3). R7041(∆Us3) virions accumulate in the PNS. The total surface area of membranes needed for envelopment was about 100 µm ^2^ which equals about 20% of the nuclear surface. Nuclear membrane folds form complicated structures. Therefore, we imaged an area of folds of the INM on serial sections employing the FIB-SEM technology. 3D reconstruction revealed that the INM folds to complicated structures enclosing cavities that contain virions ( Figure 6). We conclude that excessive production of membrane may counteract the dilation of nuclear pores.

### Number of nuclear pores declines during wt HSV-1 and R7041(∆Us3) infection

**Figure 7.**
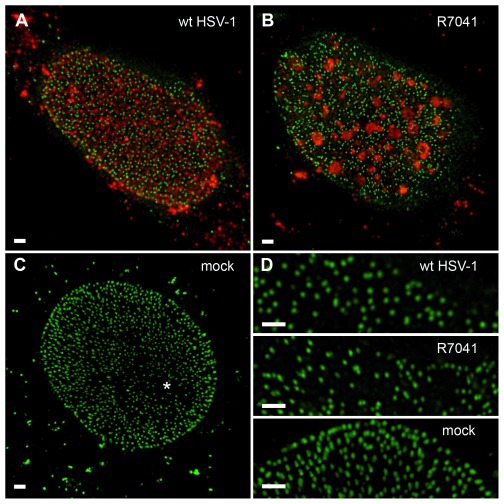
Stimulated emission depletion (STED) images of nuclei at 16 hpi with wt HSV-1 ( **A**), R7041(∆Us3) ( **B**) or mock ( **C**). The same is displayed at higher magnification in panel **D**. Nuclear pores (green), imaged by STED, are labeled with Mab414 and Alexa 532 as secondary antibody. The viral protein VP16 (red) was labeled with a polyclonal antibody and Alexa 488 as secondary antibody. In the mock infected cells ( **C**) a region devoid of pores, due to the uneven surface of a nucleus, can be observed (asterisk). Note the focal distribution of VP16 after R7041(∆Us3) infection. Bar 1µm.

**Figure 8.**
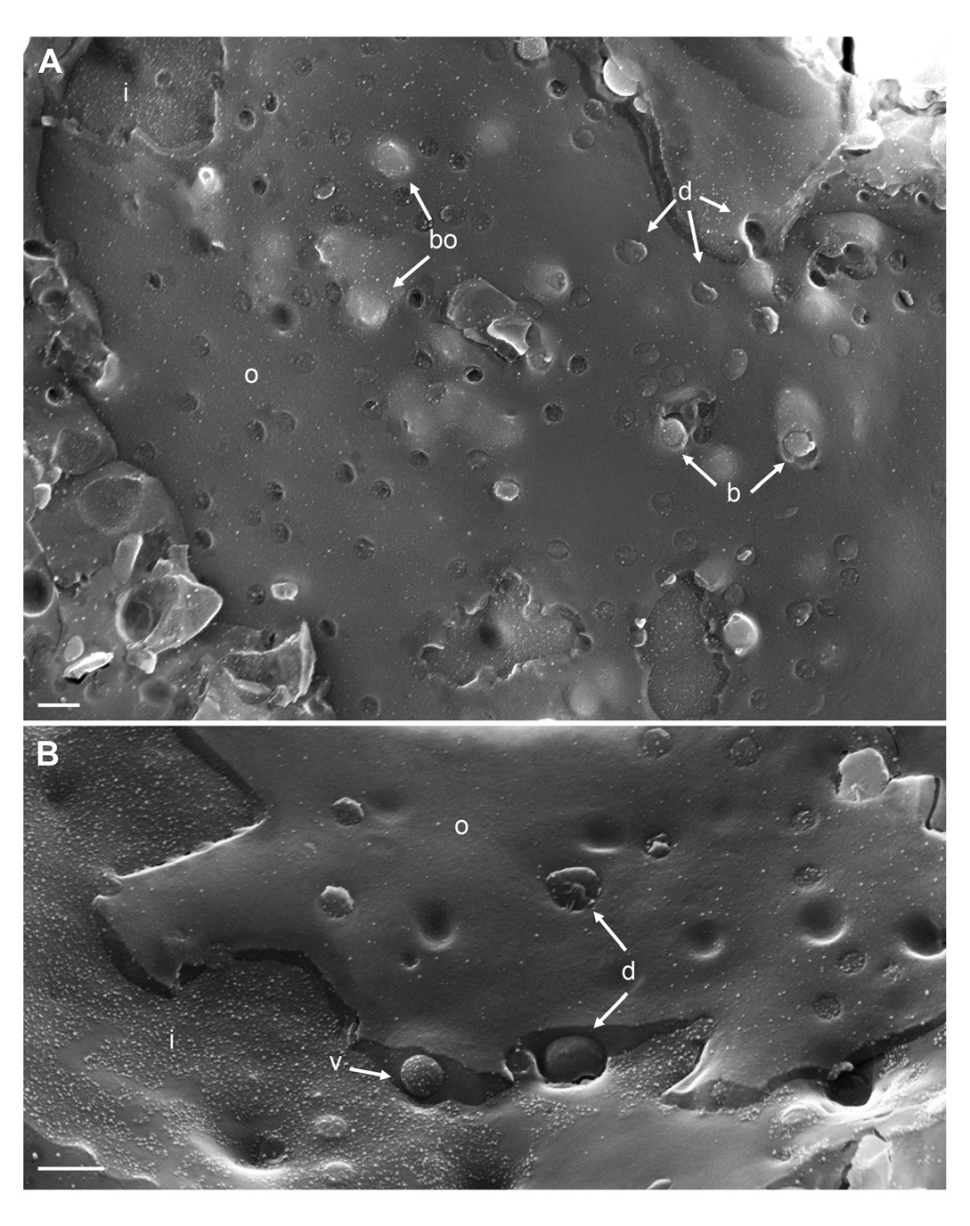
Cryo-field emission scanning electron microscopy (cryo-FESEM) images of the nuclear surface at 12 hpi with wt HSV-1 demonstrating in ( **A**) budding capsids (bo) under the outer nuclear membrane (ONM) (o) and at two sites where the ONM has been focally removed (b) during fracturing, as well as dilated nuclear pores (d) with or without protruding material. ( **B**) shows a virion (v) in the Perinuclear space (PNS), and a dilated nuclear pore (d) at the inner nuclear membrane (INM) (i) and at the ONM both occupied by protruding material. Bars 200 nm.

The number of nuclear pores was counted on cryo-FESEM images of 10 nuclei harvested at 16 hpi. Then, the mean number of nuclear pores was expressed per mean nuclear surface area ( Figure 5B) calculated from the three axes measured on confocal images. The total pore number was significantly lower (p<0.001) in cells infected with any virus compared to mock-infected cells ( Figure 5C). This was probably due to areas devoid of nuclear pores after infection ( Figure 2 and Figure 8). Confocal microscopy of Mab414 stained nucleoporins revealed also statistically significant lower numbers of (p<0.001) NPCs after infection with R7041(∆Us3), wt HSV-1 or R2641 compared to the pore number in mock infected cells ( Figure 5C). To ascertain whether resolution power of confocal microscopy was sufficient for accurate determination of pore numbers ( Wild *et al.*, 2009), we visualized nuclear pore distribution by gSTED ( Figure 7). Quantitation of nuclear pores on 3 nuclei per group revealed that the mean total number of nuclear pores per mean nuclear surface was almost equal after R7041(∆Us3) infection, 12% lower after wt HSV-1 infection but 5% higher in mock infected cells ( Figure 5C). We assume that pore numbers were overestimated to some extent in SEM images. However, determination of pore numbers in confocal images and STED images is considered very likely to result in some underestimation. Minimal interpore distance was less than 30 nm after wt HSV-1 infection, and less than 7 nm in mock infected cells ( Wild *et al.*, 2009). Lateral resolution of STED is 50 nm. Nuclei and, consequently, the nuclear surface area expand during HSV-1 infection ( Simpson-Holley *et al.*, 2005; Sutter *et al.*, 2012). Phospholipid biosynthesis is induced by HSV-1 contributing to nuclear membrane enlargement ( Sutter *et al.*, 2012). From the nuclear surface devoid of pores and nuclear membrane proteins we conclude that nuclear membranes enlarge upon HSV-1 infection, but pore formation is delayed or inhibited, and insertion of host cell membrane proteins ceased. Instead viral proteins are inserted leading to fundamental changes of nuclear membranes protein composition ( Johnson & Baines, 2011).

### Capsids bud at the inner and outer nuclear membranes

**Figure 9.**
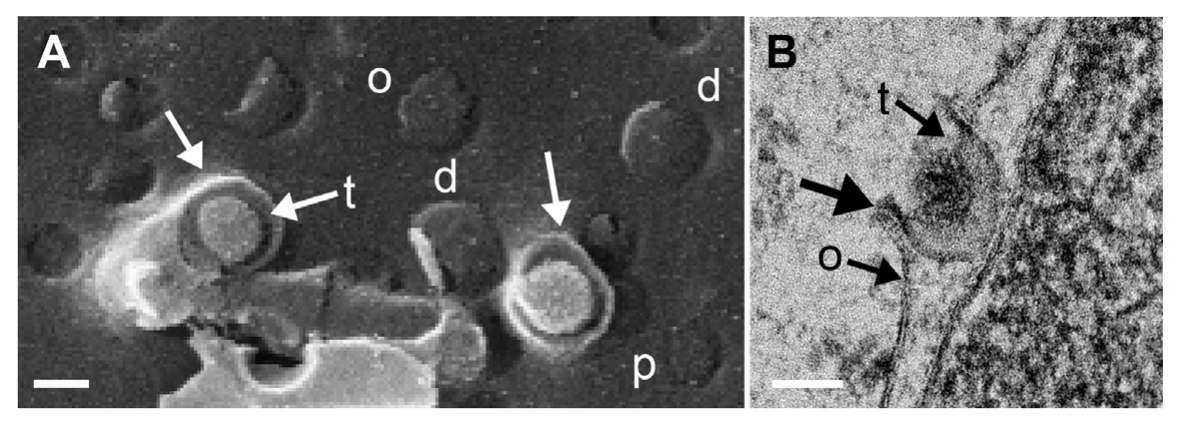
( **A**) Scanning electron microscopy (SEM) image of 2 budding capsids at the outer nuclear membrane (ONM) (o) close to normal (p) and dilated (d) pores. The ONM forms folds (arrows) that derive by the capsids being pushed towards the perinuclear space (PNS) whilst the ONM is forced to cover the capsids. The space between ONM and capsid is filled with tegument (t) ( **B**) Transmission electron microscopy (TEM) image of budding capsid at the ONM (o). Note the curvature (thick arrow) that is typical for budding, the tegument (t), which starts to be deposited between the budding front and the capsid, and the sharp bending of the ONM, which turns into the viral envelope that contains the budding proteins UL31 and UL34. Bars 100 nm.

**Figure 10.**
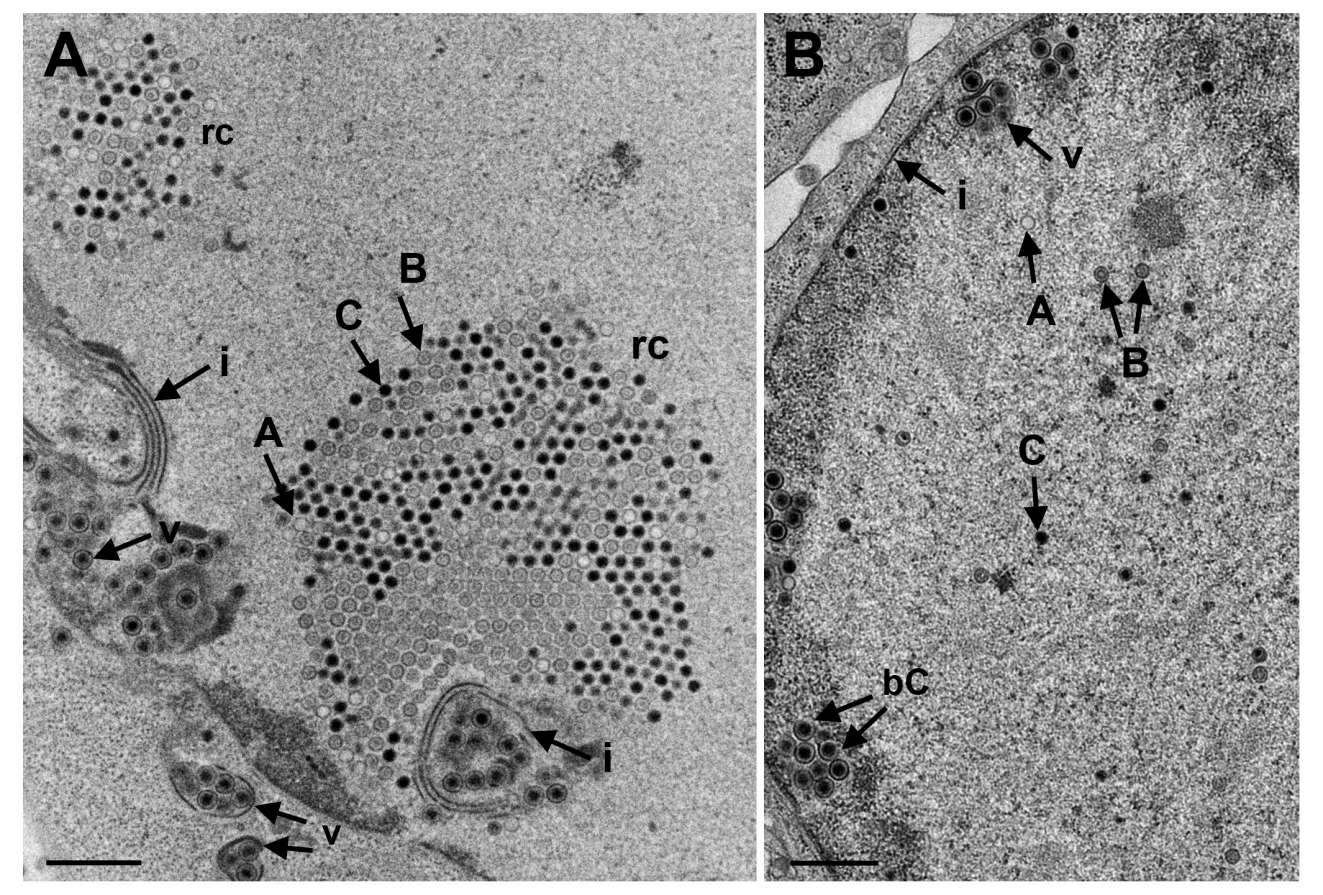
Transmission electron microscopy (TEM) images of nuclei of Vero cells at 20 hpi with R7041(∆Us3). ( **A**) RCs (replication centres) contain hundreds of B-capsids (with scaffold) and C-capsids (with DNA) as well as a few A-capsids (empty). Virions (v) accumulate in invaginations of the inner nuclear membrane (INM) (i) which had formed multiple folds. ( **B**) A-, B- and C-capsids are scattered throughout the nucleus. Two C-capsids (bC) bud at the membrane of an invagination. Bars 500 nm.

Capsids overcome the nucleocytoplasmic barrier by budding at the INM acquiring tegument and envelope. The result is a fully enveloped virion in the PNS ( Figure 8 and Figure 10). In cryo-FESEM images, budding capsids appear as spheres covered with bright dots (probably representing spikes) at the INM or in the PNS. They look like bulges when they are covered by the ONM, as clearly apparent when the ONM is partially removed. During budding, the capsid pushes the INM into the PNS whilst at the periphery the INM is pulled behind the budding capsid for fission to give rise of a virion with an electron dense envelope located in a deep indentation ( Figure 8) readily seen on the INM in cryo-FESEM images. The indentations at the ONM could be interpreted as late stages of fusion of the viral envelope with the ONM after release of capsid and tegument into the cytoplasm ( Skepper *et al.*, 2001). However, the process at the ONM takes place even in the absence of fusion proteins gB/gH ( Farnsworth *et al.*, 2007) discussed in detail by ( Wild *et al.*, 2018). Therefore, this process is budding rather than fusion, and hence, the indentations represent initial stages of budding capsids from the cytoplasm into the PNS. The phenotypes of the virus translocation across the ONM shows all characteristics of budding ( Figure 9) as discussed in detail ( Leuzinger *et al.*, 2005; Wild *et al.*, 2005; Wild *et al.*, 2012b). The budding process at the ONM was described for the first time in baby hamster kidney cells infected with herpes simplex virus strain H4 ( Darlington & Moss, 1968).

### Release of R7041(∆Us3) nuclear capsids is reduced

**Figure 11.**
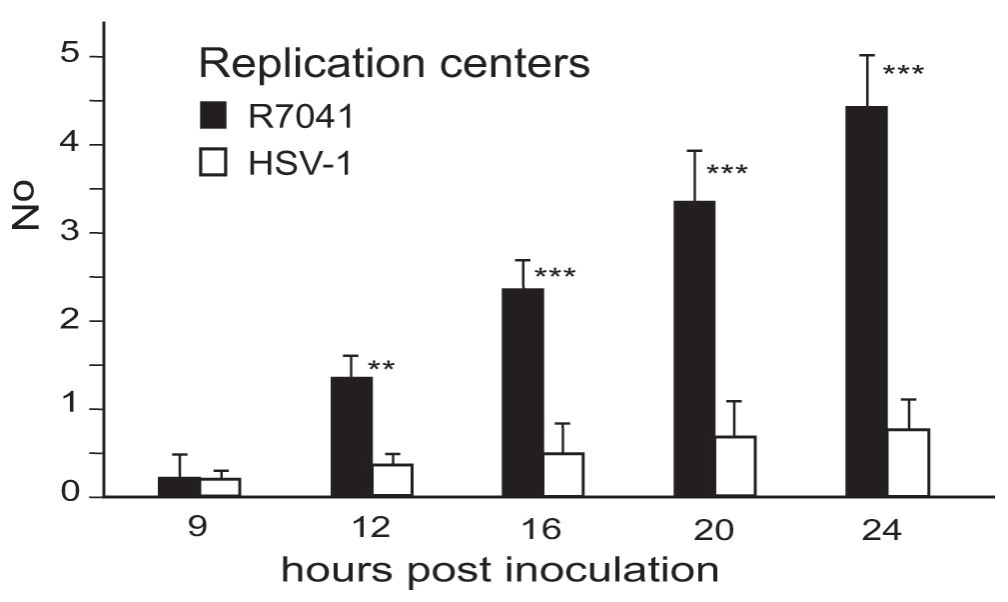
Mean number and standard deviation of replication centres (RCs) containing capsids expressed per nuclear profile. Level of significance: **p<0.001, ***p<0.0001 compared to wt HSV-1, n=5.

**Figure 12.**
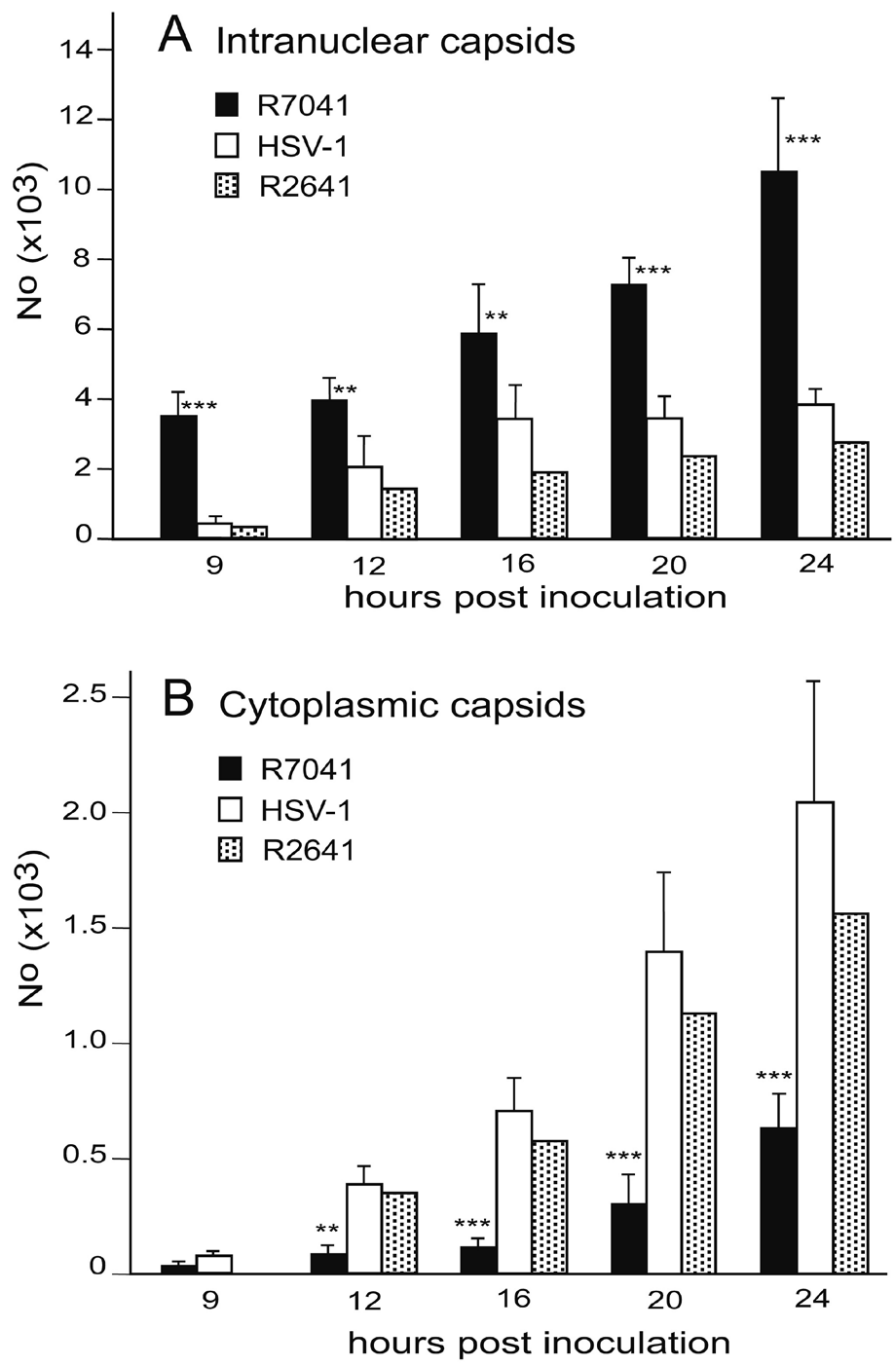
Mean number and standard deviation of capsids in randomly selected nuclei in R7041(∆Us3), R2641 or wt HSV-1 infected cells calculated per mean nuclear volume ( **A**), and of capsids in the cytoplasm including capsids free in the cytoplasmic matrix and capsids budding at Golgi membranes. Level of significance: **p<0.001, ***p<0.0001 compared to wt HSV-1 or repair mutant R2641, n=5.

**Figure 13.**
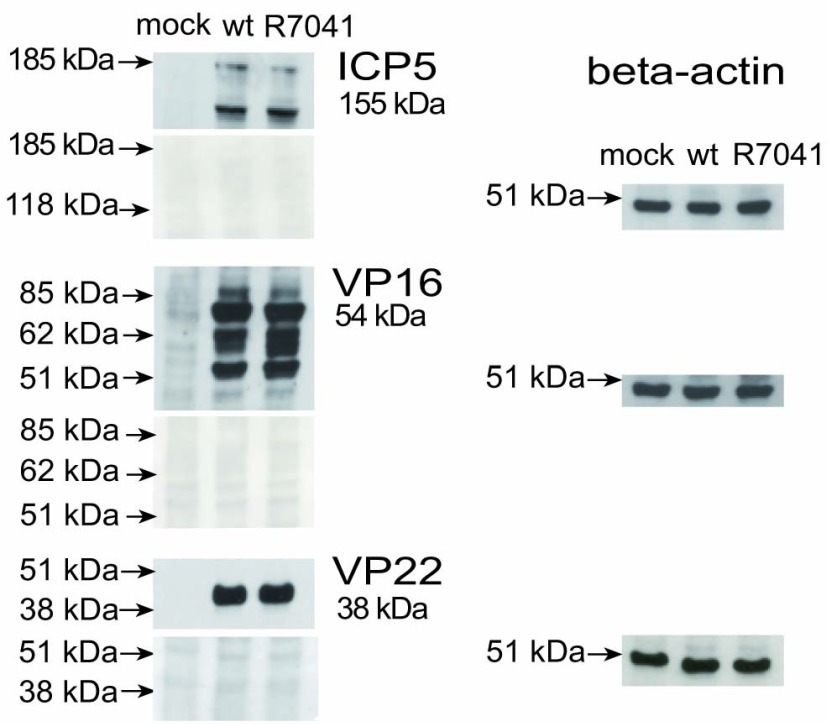
Western blots for viral proteins ICP5, VP16 and VP22 of mock, wt HSV-1 or R7041(∆Us3) infected cells harvested at 24 hpi. Beta actin staining served as loading control.

For release into the cytoplasm, capsids need to be transported from the RCs to the nuclear periphery. UL31 and UL34 have been shown to be responsible for intranuclear capsid transportation ( Simpson-Holley *et al.*, 2004). Function of UL31 and UL34 depends on phosphorylation by the Us3 kinase ( Mou *et al.*, 2009; Ryckman & Roller, 2004). Therefore, we compared the number of RCs and of capsids ( Figure 10), including A-capsids (empty capsids), B-capsids (scaffold containing capsids), and C capsids (DNA containing capsids) ( Tandon *et al.*, 2015) in 10 randomly selected Vero cells infected with R7041(∆Us3) or wt HSV-1 from 5 independent experiments. In R7041(∆Us3) infected cells, the mean number of RCs per nuclear profile was 0.3 (±0.3) at 9 hpi, and 4.5 (±0.8) at 24 hpi ( Figure 11). In contrast, the number of RCs was lower than 1 per nuclear profile at any time point after inoculation with wt HSV-1 or the repair mutant R2641. The number of intranuclear capsids dispersed throughout the nucleus was significantly higher at any time point after infection with R7041(∆Us3) compared to wt HSV-1 or the repair mutant R2641 ( Figure 12A), reaching a maximum of 10,500 (±2250) per mean nuclear volume at 24 hpi with R7041(∆Us3). Interestingly, the number of wt HSV-1 capsids increased up to 3,500 (±800) by 16 hpi, and remained constant thereafter. The higher number of RCs and capsids in R7041(∆Us3) infected cells may be due to enhanced assembly or inhibited release into the cytoplasm. Therefore, we harvested virus particles from cell cultures at 24 hpi for immunoblotting. Western blots probed with monoclonal antibodies against the capsid protein ICP5 did not reveal any obvious differences between R7041(∆Us3) and wt HSV-1 ( Figure 13). We thus assume that the higher number of RCs and of capsids is more likely the result of impeded release than of enhanced synthesis and assembly.

### Capsid release via impaired nuclear envelope is declined in the absence of Us3

Capsids gain access to the cytoplasmic matrix via impaired NE ( Borchers & Ozel, 1993; Grimm *et al.*, 2012; Klupp *et al.*, 2011; Leuzinger *et al.*, 2005; Maric *et al.*, 2014; Schulz *et al.*, 2015; Wild *et al.*, 2005; Wild *et al.*, 2012b) that starts by impairment of nuclear pores. Impairment of nuclear pore is shown in Figure 3. Capsids are not released from the PNS into the cytoplasmic matrix in the absence of Us3 ( Reynolds *et al.*, 2002; Wisner *et al.*, 2009). Nonetheless, quantitative electron microscopic analysis revealed R7041(∆Us3) capsids in the cytoplasm though at a reduced number compared to wt HSV-1 ( Wild *et al.*, 2015). As shown in Figure 12B, the number of R7041(∆Us3) capsids including those in the cytoplasmic matrix and in the process of budding at membranes was significantly lower compared to wt HSV-1 capsids at any time after 12 hpi. It was postulated that gB is not phosphorylated in the absence of the Us3 gene, and hence, the viral envelope cannot fuse with the ONM. ( Wisner *et al.*, 2009). More important, the viral envelope does not fuse with the ONM at all as discussed above. Therefore, we conclude that capsids gain access to the cytoplasm via impaired nuclear pores, and that R7041(∆Us3) capsid release declines due to reduced nuclear pore impairment.

## Discussion

### Requirement of nuclear membranes for budding

**Figure 14.**
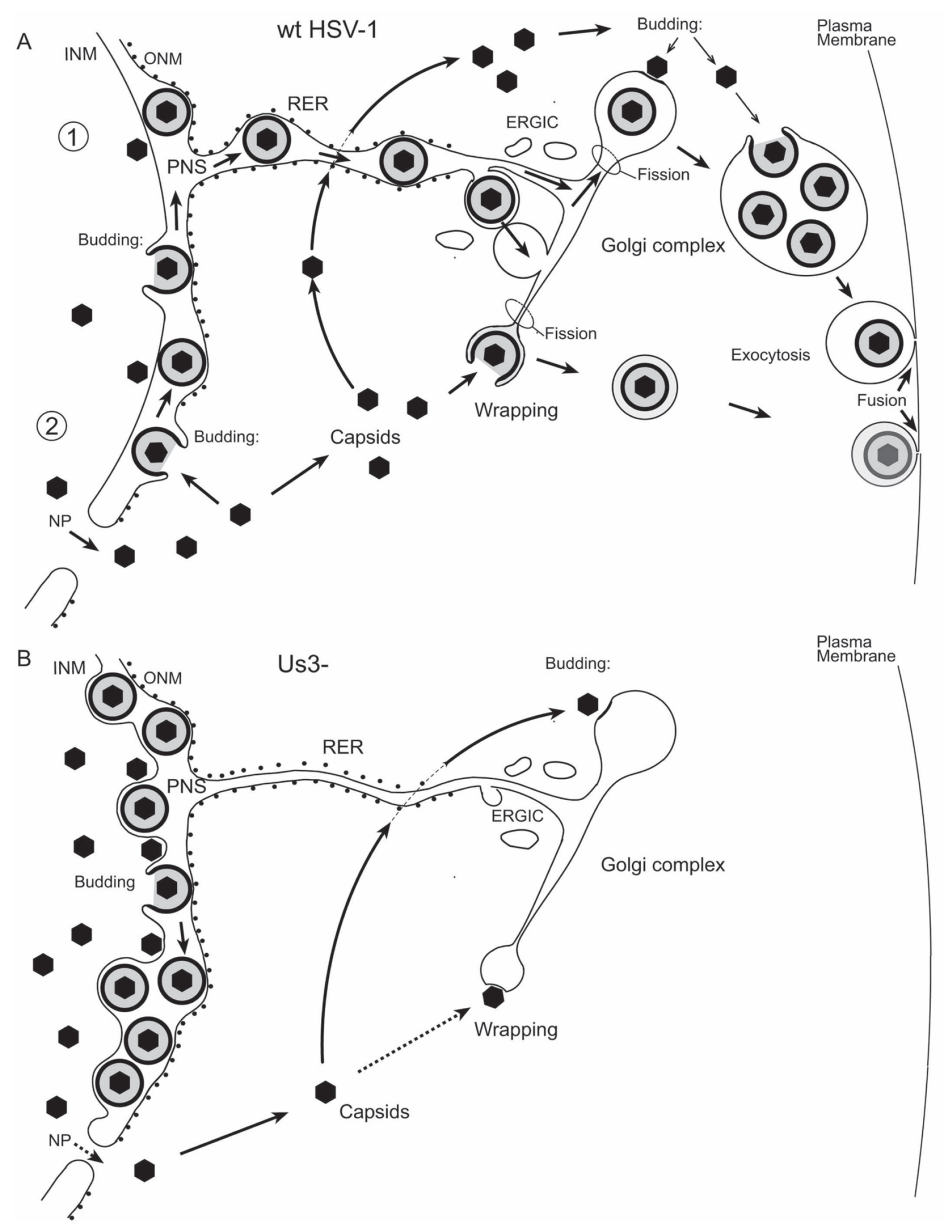
Schematic presentation of morphogenesis and intracellular transport of wt HSV-1 and Us3 deletion mutants (Us3-). ( **A**) In pathway 1, wt HSV-1 virions derived by budding at the INM are intraluminally transported from the perinuclear space (PNS) via ER-to-Golgi transitions or via ER-Golgi intermediate compartments (ERGIC), the kiss and run mechanism, into Golgi cisternae for packaging into transport vacuoles that delivers virions to the plasma membrane for exocytotic release into the extracellular space. In pathway 2, capsids released via impaired nuclear pores (NP) either bud at Golgi or vacuolar membranes into Golgi cisternae or vacuoles or are wrapped by Golgi membranes or endosomal membranes. Wrapping means budding at membranes concomitantly forming the viral envelope and the vacuolar membrane. The result is a concentric vacuole containing a single virion. ( **B**) In the absence of the Us3 gene, virions cannot be released from the PNS possibly because the intraluminal transportation route is impaired. Nevertheless, Us3 deletion mutants are infective. Nucleus to cytoplasm capsid translocation via impaired nuclear pores is inhibited. Budding into Golgi cisternae and wrapping at Golgi membranes is inhibited. 98% of enveloped virions have been shown to locate in the PNS ( Wild *et al.*, 2015).

HSV-1 replicates in the nucleus and radically alters nuclear architecture including formation of RCs, nuclear expansion and disruption of the nuclear lamina ( Simpson-Holley *et al.*, 2005). The nuclear surface expands from ~400 µm ^2^ to ~500 µm ^2^, so that ~100 µm ^2^ of membrane area must be inserted into the INM and the same amount into the ONM within the first 9 h of infection ( Sutter *et al.*, 2012). The required phospholipids are supplied by *de novo* biosynthesis. Translocation of capsids from the nucleus into the cytoplasm starts at about 8 hpi. Release of capsids by budding at the INM requires additional membranes. R7041(∆Us3) capsids bud at the INM, the resulting virions, however, cannot be transported out of the PNS ( Figure 14). Morphometric analysis revealed that ~2400 virions accumulate in the PNS of a single cell by 24 hpi ( Wild *et al.*, 2012a; Wild *et al.*, 2015) that means 98% of all enveloped R7041(∆Us3) virions produced. The diameter of a virion is 200 nm ( Grünewald *et al.*, 2003). Therefore, the surface area of 2400 virions equal an area of ~300 µm ^2^. If the idea of de-envelopment by fusion of the viral envelope with the ONM was correct ( Skepper *et al.*, 2001) the same amount of membranes used for budding at the INM would have to be inserted into the ONM. The crux is that close to 2 capsids bud simultaneously per 1 µm ^2^ nuclear surface at 10 hpi ( Wild *et al.*, 2009) demanding high dynamics in maintenance of nuclear membrane integrity. Us3 kinase down regulates phospholipid biosynthesis ( Wild *et al.*, 2012a). In the absence of the Us3 gene, the INM forms multiple folds, invaginations and evaginations due to excess biosynthesis of phospholipids. Therefore, we speculate that dilation of nuclear pores is provoked by the high demand of membranes for envelopment of budding wt HSV-1 capsids, and that pore dilation is largely prevented when membranes are over produced in the absence of the Us3 gene.

Recently, it was reported that the endosomal sorting complexes required for transport III (ESCRT-III) is responsible for scission of the viral envelope form the INM ( Arii *et al.*, 2018). ESCRT-III also is involved in maintaining INM integrity by downregulating excess INM. Interestingly, the depletion of ESCRT-III proteins induced aberrant INM proliferation in uninfected cells. In HSV-1 infected cells, virions accumulated between nuclear membranes in a similar fashion as Us3 deletion mutants. However, it has to be borne in mind that virions may accumulate in the PNS per se late in infection ( Leuzinger *et al.*, 2005). Knockdown of CD98 heavy chain and its binding partner β integrin induced invaginations of the INM that contained HSV-1 virions ( Hirohata *et al.*, 2015) resembling the phenotype of infection with Us3 deletion mutants. The question thus arises whether Us3 exerts its regulatory effect on phospholipid biosynthesis via CD98 heavy chain and/or its binding partner β integrin and/or ESCRT-III.

### Breakdown of the nuclear envelope

Breakdown of the NE in the course of herpes virus infection was reported in cells infected with Simian agent 8 ( Borchers & Ozel, 1993), PrV UL31 and UL34 recombinants ( Grimm *et al.*, 2012; Klupp *et al.*, 2011; Schulz *et al.*, 2015) and HSV-1 ( Maric *et al.*, 2014). Breakdown of the NE was considered likely to be related to dilation of nuclear pores after infection with bovine herpes virus 1 ( Wild *et al.*, 2005) and HSV-1 ( Leuzinger *et al.*, 2005). Nuclear pore dilation and nuclear membrane breakdown requires careful examination, preferably on serial sections through cells prepared by rapid freezing followed by freeze-substitution to keep membranes in place ( Wild *et al.*, 1997), to prevent loss of lipids ( Weibull *et al.*, 1984) and to improve both temporal and spatial resolution ( Mueller, 1992). Images taken from such prepared cells clearly demonstrate the difference between pore dilation and membrane breakdown ( Figure 3). Interestingly, true membrane rupture was found to be restricted to the INM. Another technique to visualize nuclei in the closest natural state is microscopy of cells in the frozen hydrated state. Cryo-FESEM revealed that the holes in the nuclear surface were clearly demarcated indicating that these holes are not the result of accidental ruptures of the INM and ONM. They are very likely dilated nuclear pores that can enlarge leading to large impaired areas of the nuclear envelope as shown for HSV-1 and BoHV-1 ( Leuzinger *et al.*, 2005; Wild *et al.*, 2005). This is in line with the statement that the initial microscopically visible event in breakdown of the nuclear envelope is dilation of nuclear pores in cells undergoing meiosis ( Terasaki *et al.*, 2001).

### Formation of nuclear pores

It was reported that disassembly of the nuclear lamina is required, and that membrane disruption is driven by microtubules ( Georgatos *et al.*, 1997). Disassembly of the lamina is induced by HSV-1 enabling successful budding of capsids at the INM ( Scott & O’Hare, 2001; Simpson-Holley *et al.*, 2005). Lamina disassembly depends on Us3 ( Bjerke & Roller, 2006; Mou *et al.*, 2007), UL31 and UL34 ( Reynolds *et al.*, 2004; Simpson-Holley *et al.*, 2005) suggesting that the Us3 kinase might be involved in NE breakdown. However, lamina disassembly is not established to cause disruption of the NE ( Prunuske *et al.*, 2006). Alternatively, breakdown of the NE has been proposed to start by disassembly of NPCs. As a consequence, nuclear pores are destabilized and expand ( Terasaki *et al.*, 2001). Therefore, we postulated that nuclear pores dilate leading to disruption when infection proceeds.

Nuclear pores are formed and NPCs are assembled during mitosis as well as in the interphase ( Doucet & Hetzer, 2010). Despite nuclear expansion in the interphase, nuclear pore number remains constant ( Doucet & Hetzer, 2010). However, the number of nuclear pores was reduced after both wt HSV-1 ( Wild *et al.*, 2009) and R7041(∆Us3) infection. The nucleoporin Nup153 was shown to be down regulated in HSV-1 infected cells ( Ray & Enquist, 2004) whereas the cellular levels of major nucleoporins remained unchanged ( Hofemeister & O’Hare, 2008; Wild *et al.*, 2009). Nuclei expand during HSV-1 infection (Simpson- Holley *et al.*, 2005; Sutter *et al.*, 2012) and during R7041(∆Us3) infection ( Wild *et al.*, 2012a). Expanding nuclei require membrane constituents for enlargement of the NE. HSV-1 has been shown to induce biosynthesis of phospholipids which are incorporated into nuclear membranes. The nuclear surface area increases by about 100 µm ^2^ within 12 hpi with wt HSV-1 ( Sutter *et al.*, 2012). The NE is a double coat. The total area of newly produce membranes equals 200 µm ^2^. The INM also provides membranes for envelopment by budding. The total requirement of membrane constituents in HSV-1 infection is reflected by the incorporation of [ ^3^H]-choline, which was twice as high by 12 hpi compared to controls. Cellular proteins are embedded in nuclear membranes. These proteins are readily visible in fracture planes of the INM ( Orci & Perrelet, 1975). Protein composition is drastically altered after HSV-1 infection, and cellular proteins are largely replaced by viral proteins ( Johnson & Baines, 2011). After infection with any of the viruses, large areas of the INM were devoid of proteins as was described also in another study employing freeze-fracture technique ( Haines & Baerwald, 1976). We thus conclude that the parts of the INM devoid of cellular proteins are the result of *de novo* synthesized phospholipids induced by HSV-1. Areas of the NE devoid of cellular proteins were also devoid of nuclear pores. The mean interpore area was almost as twice as large after HSV-1 infection. The maximal interpore area was even 10 times larger after HSV-1 infection compared to mock infection ( Wild *et al.*, 2009). Nuclear pore formation is induced by nucleoporins in the course of NPC assembly ( Fichtman *et al.*, 2010; Prunuske & Ullman, 2006) in cells undergoing mitosis. Areas of the NE devoid of cellular proteins and nuclear pores, hence, suggest that formation of nuclear pores ceased after infection with HSV-1.

### Nucleus to cytoplasm capsid translocation

R7041(∆Us3) capsids are retained within the nucleus to a much larger extent than wt HSV-1 capsids. RCs, the site of capsid assembly, are surrounded by a chromatin layer, which becomes reorganized in wt HSV-1 infection, enabling spread of capsids to the nuclear periphery. There they gain direct access to the INM because the nuclear lamina underlying the INM is disrupted ( Scott *et al.*, 2001; Simpson-Holley *et al.*, 2004). In the absence of UL31 and UL34, reorganization of the chromatin layer around RCs and disruption of the nuclear lamina does not take place. Us3 kinase functions in association with phosphorylation of UL31/UL34 ( Poon *et al.*, 2006; Reynolds *et al.*, 2001; Reynolds *et al.*, 2002; Simpson-Holley *et al.*, 2004) that led to the suggestion that in the absence of Us3 translocation of capsids to the cytoplasm is impeded. Recently, it was shown that UL31 and UL34 are the proteins responsible for budding of capsids at the INM ( Bigalke & Heldwein, 2015; Bigalke & Heldwein, 2016; Hagen *et al.*, 2015). This raises the question, whether the idea that the inability of phosphorylation of UL31/UL34 ( Reynolds *et al.*, 2001; Reynolds *et al.*, 2002) is the cause for inhibited nucleus to cytoplasm capsid translocation. The discrepancy is that capsids of UL31/UL34 deletion mutants cannot bud whereas virions of Us3 deletion mutants accumulate in the PNS. Phosphorylation of gB was also claimed to be responsible for release of HSV-1 virions out of the PNS via de-envelopment because it enables gB to act as fusion protein ( Wisner *et al.*, 2009). On the other hand, gB deletion mutants are not retained in the PNS indicating that gB is not important for virion release at all ( Farnsworth *et al.*, 2007; Klupp *et al.*, 2008). These conflicts can be explained by the erroneous interpretation of the virus transportation across the ONM to be fusion e.g. ( Mettenleiter *et al.*, 2013) ignoring the fundamentals of membrane bound transportation ( Bonifacino & Glick, 2004; Hughson, 1999; Imai *et al.*, 2006; Jahn *et al.*, 2003; Kanaseki *et al.*, 1997; Leabu, 2006; May, 2002; Mayer, 2002; Orci *et al.*, 1981; Peters *et al.*, 2004; White, 1992). The process shows all characteristics of budding. It takes place even in the absence of the fusion proteins gB/gH as obvious in Figure 2 in ( Farnsworth *et al.*, 2007) leading to accumulation of virions in the PNS. Therefore, phosphorylation of gB, UL31 and UL34 does not play any role either in budding of capsids at the INM nor in release of virions via interaction with the ONM as was suggested for PrV UL34 ( Klupp *et al.*, 2001). Us3 rather plays a significant role in intraluminal transportation ( Figure 13) of virions from the PNS into the ER ( Schwartz & Roizman, 1969) and finally into Golgi cisternae ( Leuzinger *et al.*, 2005; Wild *et al.*, 2018) in addition to its function in regulation of phospholipid-biosynthesis ( Wild *et al.*, 2012a) and apoptosis ( Benetti & Roizman, 2004; Benetti & Roizman, 2007).

## Conclusion

HSV-1 induces severe alterations in the nuclear architecture and at the nuclear periphery enabling capsid release via budding at the INM or via distortion of nuclear pores leading to breakdown of the nuclear envelope. Us3 kinase plays a significant role in alterations of the NE considering regulation of biosynthesis of phospholipids induced by HSV-1. Further investigations are needed to elucidate mechanisms leading to alterations of the NE, to understand their impact on HSV-1 envelopment, and possibly on diverse cellular functions since the NE plays other crucial roles ( Wilson & Berk, 2010) in addition to maintaining the nucleocytoplasmic barrier for controlling nuclear import and export.

## Data availability

### Underlying data

Underlying data is available from Figshare

Figshare: Dataset 1. Nuclear envelope impairment is facilitated by the herpes simplex virus 1 Us3 kinase, https://doi.org/10.6084/m9.figshare.7586153 ( Wild *et al.*, 2019)

License: CC BY 4.0
