# Supplementary material for: Nuclear envelope impairment is facilitated by the herpes simplex virus 1 Us3 kinase
Source: F1000Res. 2019 Feb 18;8:198. [Version 1] doi: 10.12688/f1000research.17802.1 (PMC6584977; doi:10.12688/f1000research.17802.1)
Supplement: Peer review history

# Peer review: Reviewer response for version 1

**DOI:** 10.5256/f1000research.19463.r49427

**Published:** 2019-06-17

**Authors:** Maria Kalamvoki

**Affiliations:** 1 Department of Microbiology, Molecular Genetics, Immunology, University of Kansas Medical Center (KUMC), Kansas City, KS, USA

This is an interesting work with many novel concepts relevant to HSV-1 primary envelopment.

 A few minor points for clarification in the discussion are listed below:

1. The authors discuss about lipid biosynthesis controlled by Us3 that results in INM production. Is this an event restricted to the nuclear membrane? Can the authors discuss whether there is also increased anterograde vesicular trafficking and whether other organelles including the Golgi network display a similar surface increase? 
2. The authors stated that fully enveloped virions are present in the PNS. In case of ΔUs3 virus these virions are fully infectious. Can the authors discuss whether the envelope virions acquire from the INM has difference versus the envelope from the TGN? According to Figure 14 there are enveloped virions that move from Golgi to the plasma membrane without losing the primary envelope. Do the authors know whether there are differences between these virions and the virions that acquire envelope from TGN or vesicular structures?
3. Is it nucleus disassembly and not rupture? Can the authors underscore that point? Do the authors think that ESCRT-III recruitment to the nuclear membrane might have some membrane repair function?

Minor points to fix in the text:

 Abstract

1. Under the results "decrement in pore numbers" somewhere the authors need to state nuclear pores.
2. Page 7, Figure 5C may be 5D in some occasions.
3. Figures could have a legend to guide people about results they observe since some time is difficult to conclude from the figure.

I have read this submission. I believe that I have an appropriate level of expertise to confirm that it is of an acceptable scientific standard.

# Peer review: Reviewer response for version 1

**DOI:** 10.5256/f1000research.19463.r46764

**Published:** 2019-04-18

**Authors:** Haidong Gu

**Affiliations:** 1 Department of Biological Sciences, Wayne State University, Detroit, MI, USA

In the manuscript “Nuclear envelope impairment is facilitated by herpes simplex virus 1 Us3 kinase”, the authors used transmission electron microscopy, Cryo-field emission scanning electron microscopy and super resolution confocal microscopy to examine the sizes and numbers of nuclear pores and nuclear membrane surface area in wild type HSV-1 or DUs3 virus infected cells. They conducted quantitative analysis and confirmed several previous observations such as nuclear membrane expands in HSV-1 infected cells, Us3 deletion leads to folds and invaginations of nuclear membrane, and Us3 kinase activity is necessary for capsid release into the cytoplasm, etc. They also found that nuclear pore number decreased HSV-1 infected cells, with more interpore distance measured, compared to that of mock infected cells. The authors generated beautiful EM images to carefully describe nuclear morphological changes that help us understand the HSV-1 egress process. Several issues need to be addressed before the manuscript can be indexed.

1. In Figure 3, the authors demonstrated nuclear envelope impairment in wt HSV-1 infected cells. In Figure 4, they described folds and invaginations of nuclear membrane in DUs3 infected cells. It would be more scientifically accurate if the authors compare both wt and DUs3 in the same TEM experiment, define the descriptive criteria for pore dilation and pore impairment, and then quantitate the pore impairment side-by-side.
2. Via Figures 1 and 2, the authors showed pore size and number in mock and HSV-1 infected cells. They went on to measure and count pore size and number for statistical analysis. However, the scale bars in Figures 1 and 2 are different. Visually it is difficult to compare the pore sizes or to understand what they meant for “large clearly confined holes”.
3. On page 7, three places in parenthesis it says “(Figure 5C)”, but it actually refers to Figure 5D. Proofreading should be done before submission. It causes a great deal of confusion.  
4. Figure 6: what does the red-orange color represent in 6B and 6C. Describe them in the figure legend.
5. Also on page 7, the last sentence of conclusion says “we conclude that nuclear membranes enlarge upon HSV-1 infection, but pore formation is delayed or inhibited, and insertion of host cell membrane proteins ceased”, the last part of “insertion of host cell membrane proteins ceased” is unfounded from the data in this manuscript.
6. On page 12, the authors showed NE impairment (Figure 3) in wt HSV-1 infected cells, and refer to previous publication that capsids are not released from PNS into cytoplasm in the absence of Us3 but capsids are found in the cytoplasm albeit in a number lower than wt infection, and then concluded DUs3 capsids enter cytoplasm via nuclear pore impairment. The part “via nuclear pore impairment” will need more evidence. Also on page 12, higher number of RC and capsids were observed in DUs3 infected cells, compared to wild type infection. How about the extracellular infectious virion production?

I have read this submission. I believe that I have an appropriate level of expertise to confirm that it is of an acceptable scientific standard.
